# MHY2233 Attenuates Replicative Cellular Senescence in Human Endothelial Progenitor Cells *via* SIRT1 Signaling

**DOI:** 10.1155/2019/6492029

**Published:** 2019-05-22

**Authors:** Shreekrishna Lamichane, Sang Hong Baek, Yeon-Ju Kim, Ji Hye Park, Babita Dahal Lamichane, Woong Bi Jang, SeungTaek Ji, Na Kyung Lee, Li Dehua, Da Yeon Kim, Songhwa Kang, Ha Jong Seong, Jisoo Yun, Dong Hyung Lee, Hyung Ryong Moon, Hae Young Chung, Sang-Mo Kwon

**Affiliations:** ^1^Convergence Stem Cell Research Center, Pusan National University, Yangsan, Republic of Korea; ^2^Molecular Inflammation Research Center for Aging Intervention, College of Pharmacy, Pusan National University, Busan, Republic of Korea; ^3^Laboratory of Cardiovascular Disease, Division of Cardiology, School of Medicine, The Catholic University of Korea, Seoul 137-040, Republic of Korea; ^4^Laboratory for Vascular Medicine and Stem Cell Biology, Department of Physiology, School of Medicine, Pusan National University, Yangsan 50612, Republic of Korea; ^5^Department of Obstetrics and Gynecology, Biomedical Research Institute, School of Medicine, Pusan National University, Busan 46241, Republic of Korea; ^6^Laboratory of Medicinal Chemistry, College of Pharmacy, Pusan National University, Busan, Republic of Korea; ^7^Research Institute of Convergence Biomedical Science and Technology, Pusan National University Yangsan Hospital, Yangsan, Republic of Korea

## Abstract

Cardiovascular diseases (CVDs) are a major cause of death worldwide. Due to the prevalence of many side effects and incomplete recovery from pharmacotherapies, stem cell therapy is being targeted for the treatment of CVDs. Among the different types of stem cells, endothelial progenitor cells (EPCs) have great potential. However, cellular replicative senescence decreases the proliferation, migration, and overall function of EPCs. Sirtuin 1 (SIRT1) has been mainly studied in the mammalian aging process. MHY2233 is a potent synthetic SIRT1 activator and a novel antiaging compound. We found that MHY2233 increased the expression of SIRT1, and its deacetylase activity thereby decreased expression of the cellular senescence biomarkers, p53, p16, and p21. In addition, MHY2233 decreased senescence-associated beta-galactosidase- (SA-*β*-gal-) positive cells and senescence-associated secretory phenotypes (SASPs), such as the secretion of interleukin- (IL-) 6, IL-8, IL-1*α*, and IL-1*β*. MHY2233 treatment protected senescent EPCs from oxidative stress by decreasing cellular reactive oxygen species (ROS) levels, thus enhancing cell survival and function. The angiogenesis, proliferation, and migration of senescent EPCs were enhanced by MHY2233 treatment. Thus, MHY2233 reduces replicative and oxidative stress-induced senescence in EPCs. Therefore, this novel antiaging compound MHY2233 might be considered a potent therapeutic agent for the treatment of age-associated CVDs.

## 1. Introduction

Cardiovascular diseases (CVDs) are the leading cause of death throughout the world [1–3]. Among various CVDs, tissue ischemia associated with stroke and coronary heart disease represents the majority [2]. Despite advancements in the field of pharmacotherapy, complete recovery from CVDs and elimination of side effects of the drugs are still major challenges. Recently, stem cell therapy is emerging as a promising strategy for the treatment of CVDs [3]. Different types of stem cells that are utilized for the treatment of ischemic heart diseases include mesenchymal stem cells (MSCs), adipose tissue-derived stem cells (Ad-MSCs), embryonic stem cells (ESCs), induced pluripotent stem cells (iPSCs), endothelial progenitor cells (EPCs), and cardiac progenitor cells (CPCs) [4].

Endothelial injury is associated with many CVDs, such as atherosclerosis, thrombosis, hypertension, and myocardial infraction. Circulating EPCs maintain endothelial integrity and neovascular functions; hence, they are candidates for cell therapy in CVDs [1, 5]. EPCs have a high capacity to migrate, proliferate, and differentiate into mature endothelial cells (ECs) [6]. EPCs can mobilize into the peripheral blood from the bone marrow and collect at injured or ischemic vascular sites to assist with vascular regeneration [7]. Senescent or impaired EPCs are risk factors of CVDs and are responsible for endothelial dysfunction [8]. With aging, stem cells lose their self-renewal capacity to proliferate, differentiate, migrate, and restitute the original function of damaged cells [5]. The functions of EPCs are age-dependent, and the capacity to repair damaged tissue varies with age. Bone marrow-derived EPCs from young rats improved angiogenesis in impaired myocardium more than those from the aged rats [7].

Sirtuin 1 (SIRT1) has been studied mostly in vascular aging and CVDs. SIRT1 is a nicotinamide adenine dinucleotide-dependent histone deacetylase, which aids in cell cycle regulation and apoptosis. SIRT1 is highly expressed in EPCs [9] and is a longevity gene that has important protective effects in CVDs [10]. An age-dependent decrease in SIRT1 levels was observed in arteries, which suggests its role in aging of the cardiovascular system. SIRT1 delays both replicative senescence and premature senescence in stem cells and differentiated cells that were exposed to oxidative stress [11]. Oxidative stress is also a key factor for endothelial senescence [8]. Beneficial effects of SIRT1 on aging *via* DNA modulation have been reported [12]. SIRT1 is normally localized in the nucleus, where it deacetylates p53, Forkhead box O (FOXO) transcription factors [13], histones, and nonhistone proteins [14]. It regulates chromatin structure, transcription, apoptosis, cell survival, DNA repair, inflammation, and oxidative stress by deacetylating numerous substrates [15]. In replicative cell senescence, the cell cycle inhibitors, p53, p21, and p16, are activated and delay cell division, [16] and the expression of cyclin D1 and cyclin E is decreased [17]. SIRT1 deacetylates p53 and reduces the ability of p53 to regulate transcription of p21, which is a cell cycle inhibitor [18]. The SIRT1 promoter binds the transcription factors FOXO3a and p53. Upon starvation, FOXO3a translocates to the nucleus and then binds the SIRT1 promoter to remove p53. Since p53 represses SIRT1 gene expression, p53 removal by FOXO3a activates SIRT1 transcription [13].

MHY2233 is a potent SIRT1 activator synthesized from 18 benzoxazole hydrochloride derivatives based on the structure of well-known SIRT1 activators, such as resveratrol and SRT1720. The binding capacity of MHY2233 to SIRT1 is 1.5-fold higher than that of resveratrol. MHY2233 was shown to suppress the acetylation of p53 in db/db mice. MHY2233 has been identified as the strongest SIRT1 activator using an *in vitro* SIRT1 activity assay, and MHY2233 induces more SIRT1 deacetylase activity than resveratrol [14]. Surprisingly, to date, there has been no study on the effects of MHY2233 on aging.

The main purpose of this study is to examine the role of the novel compound, MHY2233, in preventing vascular senescence in human EPCs. Moreover, this study is aimed at evaluating the effect of MHY2233 on the biological functions of senescent EPCs.

## 2. Materials and Methods

### 2.1. Isolation and Culture of Human EPCs

Human umbilical cord blood was provided by Pusan National University Yangsan Hospital. Mononuclear cells (MNCs) were isolated from the human umbilical cord blood by density gradient centrifugation through Ficoll (GE Healthcare, Buckinghamshire, UK). Isolated MNCs were seeded in 1% gelatin- (Sigma-Aldrich, USA) coated culture plates and cultured in endothelium growth medium-2 (EGM-2) (Lonza, USA): endothelium basal medium-2 (EBM-2) containing 5% fetal bovine serum (FBS), 1% penicillin-streptomycin (PS), human vascular endothelial growth factor (VEGF), human basic fibroblast growth factor (b-FGF), human insulin-like growth factor-1 (IGF-1), human epidermal growth factor (EGF), ascorbic acid, and GA-1000. The medium was changed daily, and colonies were cultured for further use. EPCs from passage 8 to passage 10 were used as young EPCs, and EPCs from passage 16 to passage 20 were used as senescent EPCs in the experiments.

### 2.2. Cytotoxicity Assay (Cell Viability Assay)

Passage 10 EPCs were used for the cytotoxicity assay using the D-Plus Cell Counting Kit-8 (CCK-8), lot number DI1701-01 (http://www.donginls.com). Before seeding, each 96-well plate was coated with 1% gelatin (Sigma-Aldrich, USA), incubated for 15 min at 37°C and then washed with 1x PBS (phosphate-buffered saline). Seven thousand cells were seeded per well in the required number of wells and incubated for 24 h. Then, the medium was removed and the cells were treated with different concentrations of drug for another 24 h. After that, the medium was removed and diluted CCK-8 solution was added to each well and incubated for one hour at 37°C. The absorbance was measured at a wavelength of 450 nM using a SUNRISE-absorbance microplate reader (serial number 909004125; Firmware: V 3.32 08/07/08; XFLUOR4 version V 4.51) in order to assess cytotoxicity.

### 2.3. Senescence-Associated *β*-Galactosidase (SA-*β*-gal) Assay

To measure SA-*β*-gal activity, 6-well Nunc plates, which had been coated with 1% gelatin and washed with 1x PBS, were seeded with 1 × 10^5^ cells per well. After removing the medium, the cells were stained using an x-Gal staining kit (Senescence *β*-Galactosidase Staining Kit # 9860; Cell Signaling Technology, http://www.cellsignal.com) to determine the number of senescent cells, following the manufacturer's protocol. After staining, images from five random microscopic fields were acquired by using a light microscope (OLYMPUS, Tokyo, Japan).

### 2.4. Drug Treatment

MHY2233 was provided by Prof. Hyung Ryong Moon, Laboratory of Medicinal Chemistry, College of Pharmacy, Pusan National University. The known SIRT1 activator, resveratrol (R5010; Sigma-Aldrich, USA), was used as a positive control. Similarly, the specific inhibitor of SIRT1, EX527 (E7034; Sigma-Aldrich, Ukraine), was used as a negative control. MHY2233, resveratrol, and EX527 were dissolved in dimethyl sulfoxide (DMSO; D2438; UK). Because long-term or chronic treatments with the drugs were cytotoxic, low drug concentrations were used: 10 nM of MHY2233, 100 nM of resveratrol, and 100 nM of EX527.

### 2.5. Tube Formation Assay (Angiogenesis *In Vitro*)

For the *in vitro* tube-forming assay, 7500 cells/well were seeded in 96-well plates coated with Matrigel® GFR (BD, Science, http://www.bd.com) and incubated for 6 to 8 h. The tube formation capacity of EPCs was determined by counting the number of tubes formed and by measuring the total length of the tubes formed using a microscope (OLYMPUS, Tokyo, Japan). Images were captured in one microscopic field per well under 40x magnification.

### 2.6. Quantitative Reverse Transcription-Polymerase Chain Reaction (qRT-PCR)

For determining mRNA levels, total RNA was isolated using TRIZOL® (Ambion, Life Technologies, USA), following the manufacturer's instructions. The concentration of RNA was measured by a NanoDrop™ UV spectrophotometer. One microgram of total RNA was reverse transcribed using the PrimeScript™ 1^st^ Strand cDNA Synthesis Kit (TAKARA, Japan, Cat# 6110A). SYBR® Green Real-Time PCR Mastermix (Roche, Germany) was used for determining the mRNA levels of different genes using the primers listed in Table 1. The Roche Light Cycler 96 Real-Time PCR machine was used for thermal cycling. The data were calculated using double delta Ct analysis and were normalized against control gene (GAPDH).

### 2.7. Western Blot Analysis

Total protein was extracted on ice with RIPA lysis buffer (Thermo Fisher Scientific, http://www.thermofisher.com) containing a protease inhibitor cocktail (Sigma-Aldrich, St. Louis, MO, USA), and protein concentration was quantified using a Bicinchoninic Acid Kit (Thermo Fisher Scientific, Rockford, IL, USA). Proteins were separated using 8–12% sodium dodecyl sulfate- (SDS-) polyacrylamide gel electrophoresis and were transferred to Immobilon® polyvinylidene fluoride (PVDF) membranes (Merck). The membranes were blocked with 5% skim milk for one hour at room temperature and were incubated with primary antibodies against SIRT1 (sc-74504, Santa Cruz), p53 acetylated on K382 (Ac-p53) (ab75754, Abcam), CDKN2A/p16^INK4a^ (ab108349, Abcam), p21 (ab109199, Abcam), FOXO3a (#2497, Cell Signaling), cyclin D1 (sc-8396, Santa Cruz), cyclin E (sc-481, Santa Cruz), eNOS (#9586, Cell Signaling), endothelial nitric oxide synthase (eNOS) phosphorylated on S1177 (p-eNOS) (#9571, Cell Signaling), and glyceraldehyde-3-phosphate dehydrogenase (GAPDH, sc-25778 and sc-47724, Santa Cruz) at 4°C overnight. After washing with 1x TBST (Tris-buffered saline containing 0.1% Tween 20), the membranes were incubated with secondary antibodies at room temperature for one hour. Anti-rabbit horseradish peroxidase (HRP) conjugate (ADI-SAB-300-J, EnZo) and anti-mouse HRP conjugate (ADI-SAB-100-J, EnZo) were used as secondary antibodies. After washing the membranes again with 1x TBST, the bands were visualized using Luminata Crescendo Western HRP Substrate (Millipore; Cat. No. WBLUR0500) and X-ray film. GAPDH was used as a loading control for all western blots.

### 2.8. Cell Proliferation Assay

EPCs (7000 cells/well) were seeded in 96-well plates coated with 1% gelatin and incubated for 6, 24, or 48 h. Cell proliferation was determined by WST-8 assay, using the D-Plus CCK-8 (lot number DI1701-01; http://www.donginls.com). The absorbance after treatment with CCK-8 was measured at 450 nM using a SUNRISE-absorbance microplate reader (serial number 909004125; Firmware: V 3.32 08/07/08; XFLUOR4 version V 4.51). Cell proliferation was also evaluated by cell counting after trypan blue (Welgene) staining. Senescent cells (5 × 10^4^ per well, passage 19) were pretreated with DMSO, 10 nM MHY2233, 100 nM resveratrol, or 100 nM EX527, seeded in 6-well plates, and cultured in EGM2 medium for 6, 12, 24, or 48 h. The number of unstained cells at each time point was counted after trypsinization.

### 2.9. Scratch Wound Healing Assay

Senescent EPCs (2 × 10^5^ cells/well, passage 20) were pretreated with DMSO, 10 nM MHY2233, 100 nM resveratrol, or 100 nM EX527 and seeded in 6-well plates. Cell migration was assessed by the scratch wound healing method. After 24 h of culturing, at full confluence, a linear gap in the cell monolayer was created by scratching the surface with an SPLScar Scratcher (SPL Life Science, Korea). The cells were washed to remove detached cells and incubated at 37°C. Images were captured by using a light microscope (OLYMPUS, Tokyo, Japan) at 0, 2, 4, and 6 h. The migrated area was calculated using the following formula:

(1) $$\begin{array}{c} \text{Percentage of migrated area}=\left\{\frac{\text{original scratch area}-\text{new scratch area}}{\text{original scratch area}}\right\}\times 100\%. \end{array}$$

### 2.10. Transwell^®^ Migration Assay

Transwell® migration assays were performed using 24-well Transwell® inserts (8 *μ*M pore size; Costar, USA). The upper sides of the inserts were coated with 1% gelatin and placed on a clean bench for 30 min. In each upper insert, 1 × 10^4^ cells were seeded with EBM-2 containing 2% FBS; EGM-2 containing 5% FBS, 1% PS, and other endothelial cell growth supplements was placed in each lower chamber. The cells were incubated at 37°C for 6 h. Afterward, the cells were fixed for 10–15 min in 4% paraformaldehyde. The cells were then stained with 0.5% crystal violet (25% methanol and 1% crystal violet at a ratio of 1 : 1) for one hour. The inserts were washed twice with distilled water, and the membranes were excised and mounted on glass slides. The cell migration capacity was quantified by counting the number of cells that had migrated in five random microscopic fields (100x magnification) using a microscope (AXIO Imager; M2 ZEISS).

### 2.11. SIRT1 Deacetylase Activity Assay

SIRT1 deacetylase activity was measured by using a commercial kit (SIRT1 Assay Kit CS1040; Sigma-Aldrich) following the manufacturer's protocol. In brief, 5 *μ*L NAD^+^ solution (cat. No. N1663) was added per well in white 96-well plates except for the blank wells. After adding a 5 *μ*L activator (resveratrol), 5 *μ*L inhibitor (EX527), or 5 *μ*L sample drug (MHY2233) in the respective wells, 1.5 *μ*g human SIRT1 recombinant protein expressed in *Escherichia coli* (cat. No. S8446) was added to each well. Then, a 10 *μ*L SIRT1 substrate (cat. No. S9821) was added to each well. The final volume was brought to 50 *μ*L by adding the required volume of assay buffer (cat. No. A6480). The plate was mixed in a horizontal shaker for 1 min. After incubating the plate at 37°C for 30 min, 5 *μ*L developing solution (cat. No. D5068) was added and mixed by pipetting. The plate was again incubated at 37°C for 10 min. Then, the fluorescence signal was measured at excitation/emission wavelengths of 355/460 nM using a Multilabel Plate Reader (VICTOR3). Finally, the SIRT1 deacetylase activity was calculated using a standard curve.

### 2.12. Immunocytochemistry

Senescent EPCs (5000 cells/well, passage 17) that had been pretreated with DMSO or 10 nM MHY2233 were seeded on glass coverslips in 24-well tissue culture plates. The cells were fixed with 4% formaldehyde for 10 min and permeabilized with 0.1% Triton X-100 for 5 min. Then, the fixed cells were blocked for one hour at room temperature using 10% normal goat serum containing 0.3 M glycine in PBS. After washing with 1% BSA in PBS, the cells were incubated with specific primary antibody (1 : 100 dilution) overnight at 4°C. The cells were washed with 1% BSA in PBS four times and incubated with Alexa Fluor® 594 goat anti-mouse or Alexa Fluor® 594 goat anti-rabbit secondary antibody (Invitrogen) for 2 h at room temperature in the dark. After washing, the nuclei were stained with 4′,6-diamidino-2-phenylindole (DAPI, 1 *μ*g/mL). After washing four times, the coverslip was removed and mounted on a glass slide using Prolong® Diamond Antifade Mountant (Ref. p36961, Molecular Probes, Life Technologies). Confocal images were acquired using a Confocal Laser Scanning Microscope-KM (ZEISS LSM 700).

### 2.13. Cell Cycle Analysis

For cell cycle analysis, passage 18 senescent EPCs pretreated with DMSO, MHY2233, resveratrol, or EX527 were trypsinized when cell confluence was approximately 60–70% and collected. The harvested cells were washed with 1x PBS, fixed in 70% cold ethanol at 4°C overnight, washed with FACS buffer (2% FBS and 2 mM EDTA in PBS), and stained with Hoechst 33342 (1 *μ*g/mL) and Pyronin Y (1 *μ*g/mL) for 20 min at room temperature in the dark. Cell cycle distribution was then analyzed by flow cytometry (BD FACSCanto M). Cell cycle distributions were assessed by using FlowJo single cell analysis software (V10.3, USA).

### 2.14. Cellular Reactive Oxygen Species (ROS)

To determine cellular ROS levels, EPCs were treated with DMSO, 10 nM MHY2233, 100 nM resveratrol, or 100 nM EX527 for 24 h and then treated with 600 *μ*M H_2_O_2_ for 30 min. Afterward, the cells were detached with trypsin and washed with FACS buffer. Then, the cells were stained with 10 *μ*M 6-carboxy-2′,7′-dichlorodihydrofluorescein diacetate (carboxyl H2DFFDA) in FACS buffer for 30 min at 37°C and washed with FACS buffer three times. ROS levels were determined using fluorescence-activated cell sorting (FACS; BD Accuri C6).

### 2.15. Annexin V and Propidium Iodide (PI) Staining Assay

To measure the percentage of apoptotic cells, we harvested the cells and washed them twice with cold PBS. Then, the cells were suspended in 1x binding buffer followed by the addition of 5 *μ*L fluorescein isothiocyanate- (FITC-) annexin V and 5 *μ*L PI. After gentle vortexing, the cells were incubated for 5 min at room temperature in the dark. Then, 400 *μ*L of 1x binding solution was added to each tube. Finally, the percentage of apoptotic cells was measured by FACS (BD Accuri C6) and separated as live cells (FITC-annexin V^−^/PI^−^), apoptotic cells (FITC-annexin V^+^/PI^−^), and dead cells (FITC-annexin V^+^/PI^+^).

### 2.16. Statistical Analysis

Data are presented as means ± standard error of the mean (SEM) of at least three replicates and analyzed by using GraphPad Prism 5.0 software. Unpaired Student's *t*-tests were used to compare between two groups, and one-way analysis of variance (ANOVA) was used to analyze the difference among more than two groups using Tukey-Kramer multiple post hoc comparisons. A *p* value ≤ 0.05 was considered statistically significant (^∗^ *p* < 0.05, ^∗∗^ *p* < 0.005, and ^∗∗∗^ *p* < 0.0005).

## 3. Results

### 3.1. Characterization of Replicative Senescent EPCs

**Figure 1:**
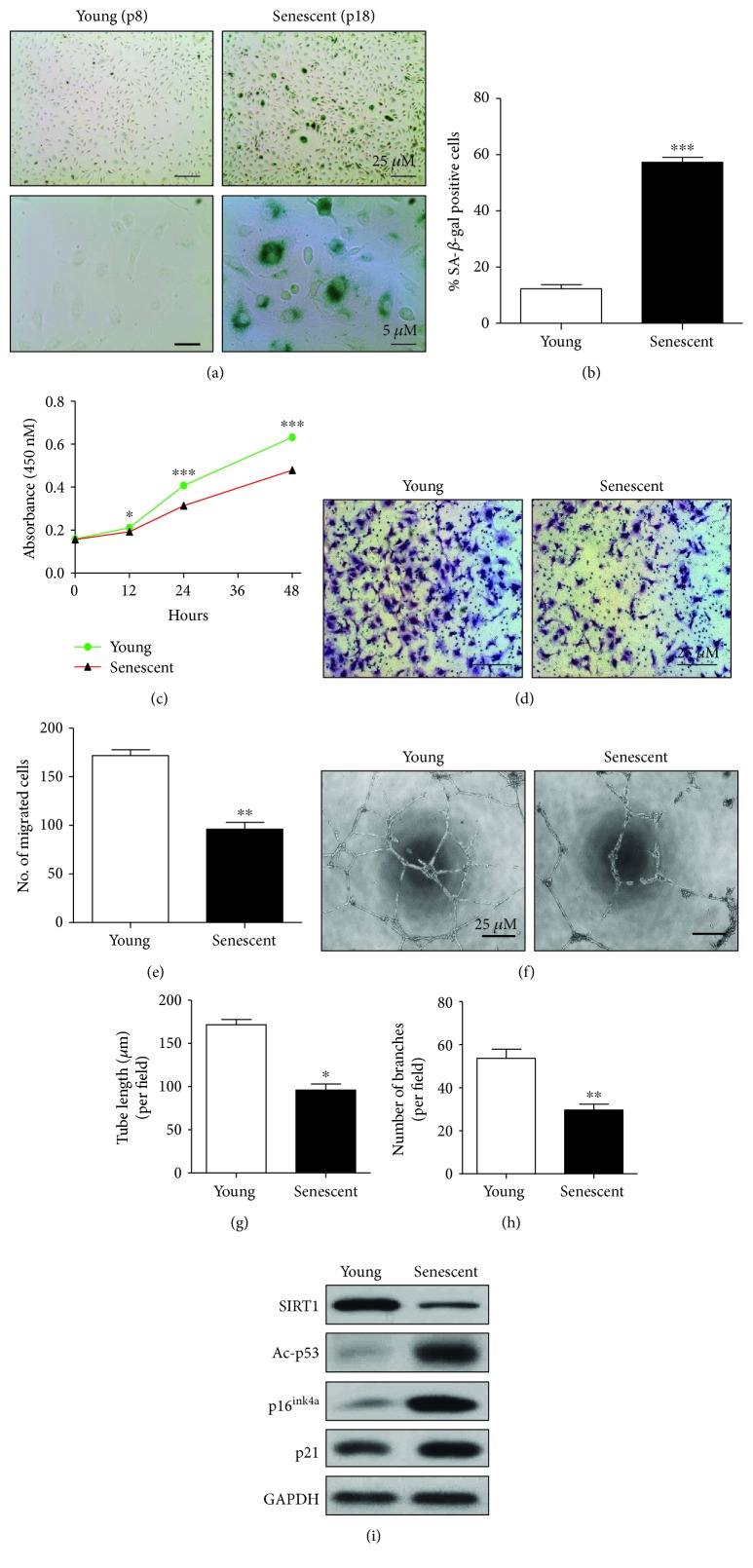
Characterization of senescent EPCs: (a) passage 8 EPCs were considered young, and passage 18 EPCs were considered senescent. Young cells and senescent cells were cultured in medium, and SA-*β*-gal staining was performed. Representative images of SA-*β*-gal-stained young and senescent EPCs are shown. SA-*β*-gal-positive cells are stained a green color. Scale bars: 25 *μ*M and 5 *μ*M. (b) Quantification of SA-*β*-gal-positive cells from three replicates (*n* = 3 per group). (c) Young and senescent EPCs were seeded in 96-well plates, and cell proliferation capacity was determined using the WST-8 assay. Absorbance was measured at 0, 12, 24, and 48 h at 450 nM wavelength (*n* = 6 per group). (d) Young and senescent cells were seeded in the upper chamber of a Transwell® culture plate. After 6 h, migrated cells were stained and images were taken under a microscope at 100x magnification (*n* = 3 wells per group). (e) Quantification of the number of migrated cells among young and senescent EPCs using ImageJ software. (f) For tube formation capacity, young and senescent EPCs were seeded in 96-well plates containing Matrigel®. After 6 h, representative images were taken under a microscope at 40x magnification (*n* = 3 per group). Scale bars: 25 *μ*M. (g) Quantification of the tube length of branches per field in *μ*M and (h) number of tube branches per field using ImageJ software (*n* = 3). (i) Cells were harvested, and total cell lysates were subjected to western blot analysis for protein expression of SIRT1, acetylated p53, p16^INK4a^, and p21 for young and senescent EPCs. ^∗^ *p* < 0.05, ^∗∗^ *p* < 0.005, and ^∗∗∗^ *p* < 0.0005*vs.* young EPCs by unpaired Student's *t*-test.

To investigate the characteristic features of young EPCs and senescent EPCs, SA-*β*-gal activity, senescence marker proteins, and mRNA expression levels between young and senescent EPCs were evaluated. The number of SA-*β*-gal-positive cells was significantly higher in senescent EPCs than in young EPCs (Figures 1(a) and 1(b)). To evaluate the difference between functional activities of young and senescent EPCs, cell proliferation, Transwell® migration, and tube formation assays were performed. The cell proliferation capacity of young EPCs (Figure 1(c)) and the number of migrating cells in the Transwell® were significantly increased compared to that of senescent EPCs (Figures 1(d) and 1(e)). Moreover, the tube-forming capacity of young EPCs in Matrigel® was significantly higher compared to that of senescent EPCs (Figure 1(f)). Likewise, the tube length and number of branches formed in Matrigel® were markedly decreased in senescent EPCs (Figures 1(g) and 1(h)). We also checked the expression levels of the senescence-associated markers, SIRT1, acetylated p53, p16^INK4a^, and p21. The protein expression of SIRT1 was decreased in senescent EPCs whereas that of Ac-p53, p16^INK4a^, and p21 was increased (Figure 1(i)). Similarly, the mRNA level of SIRT1 was found to be decreased markedly, but the mRNA level of p53, p21, and p16 was significantly increased in senescent EPCs when compared with that of young EPCs (Supplementary Figure S1). Thus, these results clarify the characteristic differences between young and senescent EPCs and suggest that the functional activities of EPCs decrease with aging or senescence.

### 3.2. SIRT1 Deacetylase Activity of the Novel Antiaging Compound MHY2233

**Figure 2:**
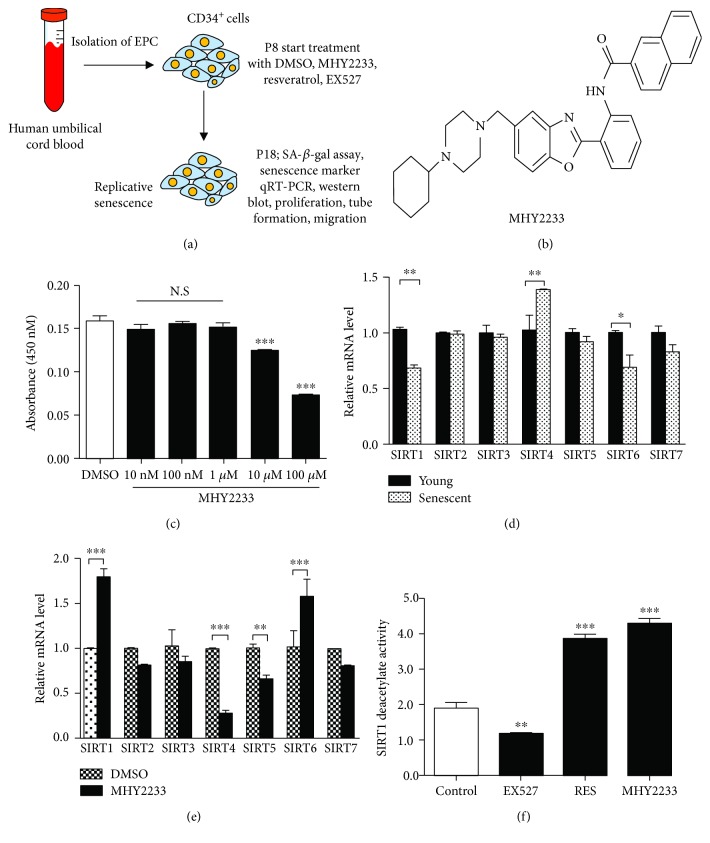
SIRT1 deacetylase activity of MHY2233: (a) schematic diagram to show isolation of EPCs from the human umbilical cord blood, treatment with drugs, and other basic experimental designs. (b) Chemical structure of MHY2233. (c) EPCs were seeded in 96-well plates and treated with different concentrations of MHY2233 for 24 h (*n* = 6 per group). Cytotoxicity assay of MHY2233 at different concentrations was determined using WST-8 assay (*n* = 6 per group). (d) Relative mRNA levels of SIRT1 to SIRT7 in young and senescent EPCs were evaluated by qRT-PCR (*n* = 3 per group). (e) Relative mRNA expression of SIRT1 to SIRT7 in senescent EPCs treated with DMSO and MHY2233 (*n* = 3 per group). (f) SIRT1 deacetylase activity was performed using a commercially available kit. Measurement of SIRT1 deacetylase activity of MHY2233 along with positive and negative controls (*n* = 6 per group). ^∗^ *p* < 0.05, ^∗∗^ *p* < 0.005, and ^∗∗∗^ *p* < 0.0005*vs.* DMSO (control) and indicated groups by a one-way ANOVA test.

First, we isolated EPCs from the human umbilical cord blood; the experimental design is outlined in Figure 2(a). The novel compound MHY2233 was synthesized as an activator of SIRT1 based on the structure of resveratrol and SRT1720 (Figure 2(b)) [14]. Then, we assessed the cytotoxicity of MHY2233 toward EPCs. The cells were treated with different concentrations of MHY2233 for 24 h. MHY2233 was not toxic to the cells up to 1 *μ*M, but concentrations greater than 1 *μ*M were found to be cytotoxic (Figure 2(c)). We also checked the cytotoxicity of resveratrol (a known SIRT1 activator) and EX527 (a known SIRT1 inhibitor) in EPCs and found that resveratrol and EX527 were not toxic up to 10 *μ*M and 5 *μ*M, respectively (Supplementary Figure S2). Then, we quantified the mRNA levels of different sirtuin family members (SIRT1 to SIRT7) in young and senescent EPCs and found that SIRT1 and SIRT6 mRNA decreased significantly in senescent EPCs, whereas SIRT4 mRNA increased significantly (Figure 2(d)). After treating the cells with 1 *μ*M MHY2233, we found that the mRNA levels of SIRT1 and SIRT6 were increased, whereas those of SIRT4 and SIRT5 were significantly decreased compared to the corresponding levels in the DMSO group (control). There was no significant change in the mRNA levels of SIRT2, SIRT3, and SIRT7 (Figure 2(e)). Furthermore, we measured SIRT1 deacetylase activity after MHY2233 treatment using a commercially available kit and found that MHY2233 induced more SIRT1 deacetylase activity than the positive control, resveratrol (Figure 2(f)). Thus, these results suggest that MHY2233 is a new potent compound that activates SIRT1.

### 3.3. Rescue of Replicative Senescence in EPCs by MHY2233 Treatment *via* SIRT1 Signaling

**Figure 3:**
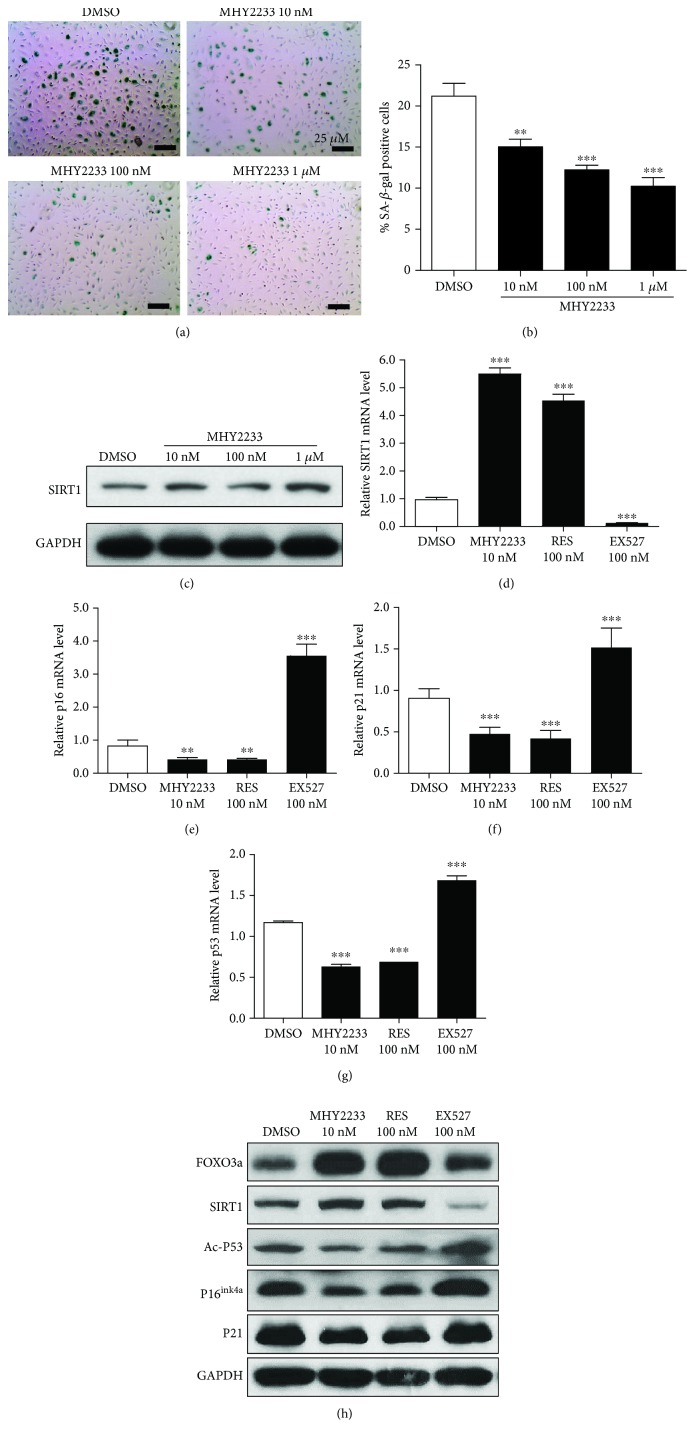
Roles of MHY2233 in SIRT1 signaling and rescue of replicative senescence in EPCs: (a) EPCs were cultured with DMSO or MHY2233 (10 nM, 100 nM, or 1 *μ*M) for replicative senescence, and SA-*β*-gal staining was performed. Representative images of SA-*β*-gal stain in senescent EPCs. Cells stained green are SA-*β*-gal-positive cells. (b) Quantification of SA-*β*-gal-positive cells (*n* = 3 per group). (c) Cells were harvested, and total cell lysates were used in western blot analysis for protein expression of SIRT1 in senescent EPCs upon treatment with different concentrations of MHY2233. (d) After chronic treatment of senescent EPCs with DMSO, 10 nM MHY2233, 100 nM resveratrol, or 100 nM EX527, mRNA and total protein were isolated. Measurement of relative mRNA levels of SIRT1, (e) p16, (f) p21, and (g) p53 was analyzed using qRT-PCR (*n* = 3 per group). (h) Whole cell lysates were subjected to western blot analysis for determining protein levels of FOXO3a, SIRT1, acetylated p53, p16^INK4a^, and p21 in senescent EPCs. ^∗^ *p* < 0.05, ^∗∗^ *p* < 0.005, and ^∗∗∗^ *p* < 0.0005*vs.* DMSO (control) by a one-way ANOVA test.

To determine the role of MHY2233 in the replicative senescence of EPCs, senescent EPCs were treated with MHY2233 in a concentration-dependent manner and senescence markers, including SA-*β*-gal activity, were measured. The results show that MHY2233 treatment significantly decreased the percentage of SA-*β*-gal-positive cells concentration-dependently (Figures 3(a) and 3(b)). To establish the role of MHY2233 in preventing replicative senescence of EPCs, SIRT1 protein and mRNA levels were assessed in EPCs after concentration-dependent treatment with MHY2233. SIRT1 protein and mRNA levels both were upregulated with increasing concentrations of MHY2233 (Figure 3(c) and Supplementary Figure S3A). Furthermore, treatment with MHY2233 decreased the mRNA levels of p16, p53, and p21 (Supplementary Figure S3B, C, and D). Collectively, these data indicate that MHY2233 treatment increases SIRT1 gene expression and decreases senescent markers in a concentration-dependent manner, which highlights the role of MHY2233 in rescuing replicative EPC senescence.

To compare the effect of the chronic treatment of MHY2233 with that of resveratrol and EX527, EPCs were treated with low concentrations. In this experiment, the mRNA and protein levels of SIRT1 were increased by MHY2233 and resveratrol, but were decreased by EX527 (Figures 3(d) and 3(h)). The protein and mRNA levels of SIRT1 after MHY2233 treatment were greater than those after resveratrol treatment, even though the concentration of resveratrol (100 nM) was much higher than that of MHY2233 (10 nM). Similarly, the mRNA levels of p16, p21, and p53 were decreased by MHY2233 and resveratrol treatment but were increased by treatment with the SIRT1 inhibitor, EX527, when compared with that of DMSO treatment alone (Figures 3(e), 3(f), and 3(g)). The protein levels of Ac-p53, p16^INK4a^, and p21 were significantly decreased by MHY2233 and resveratrol, but as expected, they were increased by EX527. Moreover, the protein level of FOXO3a was significantly increased by MHY2233 and resveratrol, whereas it was decreased by EX527 (Figure 3(h)). Immunocytochemistry of senescent EPCs treated with DMSO and MHY2233 confirmed the respective changes in the expression of SIRT1, Ac-p53, p21, and p16^INK4a^ in both the cytoplasm and nucleus (Supplementary Figure S4). These results signify that MHY2233 decreases the replicative senescence of EPCs by stimulating SIRT1 activity and SIRT1 gene expression.

### 3.4. MHY2233 Attenuates Oxidative Stress-Mediated Senescence in EPCs

**Figure 4:**
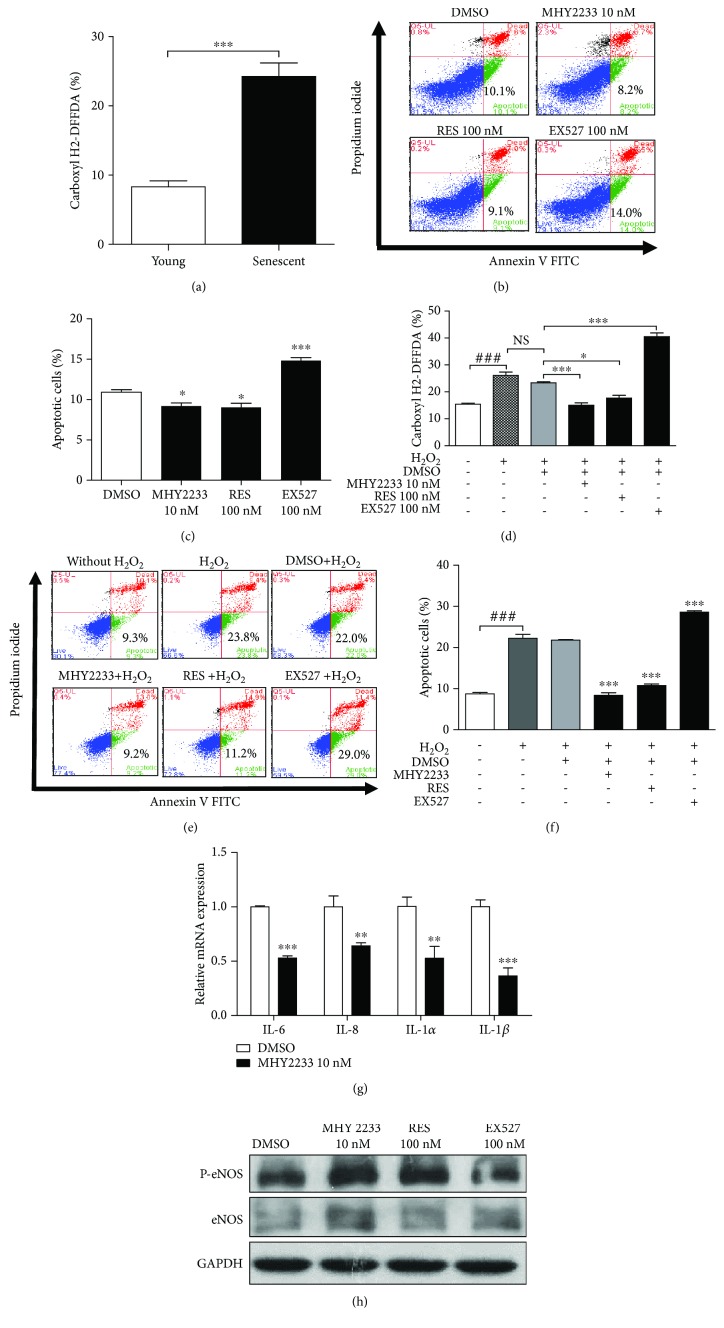
Effect of MHY2233 on oxidative stress and cell survival: (a) young and senescent EPCs were collected and stained with carboxyl H2DFFDA to determine cellular ROS levels using flow cytometry (*n* = 3 per group). (b) After chronic treatment of senescent EPCs with DMSO, 10 nM MHY2233, 100 nM resveratrol, or 100 nM EX527, the percentage of apoptotic cells was measured by annexin V/PI labeling and FACS assessment of live cells (annexin V^−^/PI^−^), apoptotic cells (annexin V^+^/PI^−^), and dead cells (annexin V^+^/PI^+^). (c) Quantification of percentage of apoptotic cells plotted in a bar graph (*n* = 3 per group). (d) Cells were treated with DMSO, 10 nM MHY2233, 100 nM resveratrol, or 100 nM EX527 for 24 h and cotreated with or without 600 *μ*M H_2_O_2_ for 30 min. Cells were collected and stained with carboxyl H2DFFDA to determine cellular ROS levels using flow cytometry (*n* = 3 per group). (e) Percentage of apoptotic cells was calculated by annexin V/PI staining and flow cytometry. (f) Percentage of apoptotic cells presented in a bar graph (*n* = 3 per group). (g) RNA was isolated, and relative mRNA levels of IL-6, IL-8, IL-1*α*, and IL-1*β* between DMSO- and 10 nM MHY2233-treated groups were determined by qRT-PCR (*n* = 3). (h) Cell lysates were used to determine protein levels of eNOS and p-eNOS in the various groups by western blot analysis. ^∗^ *p* < 0.05, ^∗∗^ *p* < 0.005, and ^###,^^∗∗∗^ *p* < 0.0005*vs.* DMSO (control) and indicated groups by a one-way ANOVA test and unpaired Student's *t*-test.

The cellular ROS levels of young EPCs and senescent EPCs were determined using carboxyl H2DFFDA staining and FACS, and we found that the senescent EPCs had significantly elevated ROS levels compared to those of the young EPCs (Figure 4(a)). To determine the effect of the long-term treatment of MHY2233, resveratrol, and EX527 on EPCs, the percentage of apoptotic cells was determined. The results show that MHY2233 and resveratrol significantly reduced the percentage of apoptotic cells, whereas the percentage was increased by EX527 (Figures 4(b) and 4(c)). EPCs were treated with H_2_O_2_ to mimic oxidative stress conditions. Cellular ROS with or without H_2_O_2_ treatment were measured. H_2_O_2_ treatment increased the cellular ROS levels in EPCs. After cotreatment of EPCs with H_2_O_2_ and DMSO, MHY2233, resveratrol, or EX527, cellular ROS levels were quantified. Cellular ROS levels decreased with MHY2233 and resveratrol treatment, but increased significantly with EX527 treatment, when compared to the levels with DMSO treatment (Figure 4(d)). The percentage of apoptotic cells was assessed after cotreatment with H_2_O_2_ and DMSO, MHY2233, resveratrol, or EX527. The percentage of apoptotic cells increased dramatically in the H_2_O_2_-treated group compared to that in the untreated group. There was no significant difference in the percentage of apoptotic cells between the H_2_O_2_-treated group and the H_2_O_2_ and DMSO-treated group. The MHY2233-treated group had fewer apoptotic cells than the resveratrol-treated group did. However, the EX527-treated group contained a markedly increased percentage of apoptotic cells (Figures 4(e) and 4(f)). This indicates that MHY2233 is more efficient in protecting EPCs from oxidative stress than resveratrol. MHY2233 treatment also decreased the mRNA levels of various SASPs, namely, IL-6, IL-8, IL-1*α*, and IL-1*β* (Figure 4(g)). MHY2233 also increased the phosphorylation of eNOS on serine 1177 (Figure 4(h)). Thus, MHY2233 is essential to protect EPCs from oxidative stress, and it prevents cell apoptosis by increasing eNOS phosphorylation and downregulating SASPs through the activation of SIRT1.

### 3.5. Effect of MHY2233 on Cell Proliferation

**Figure 5:**
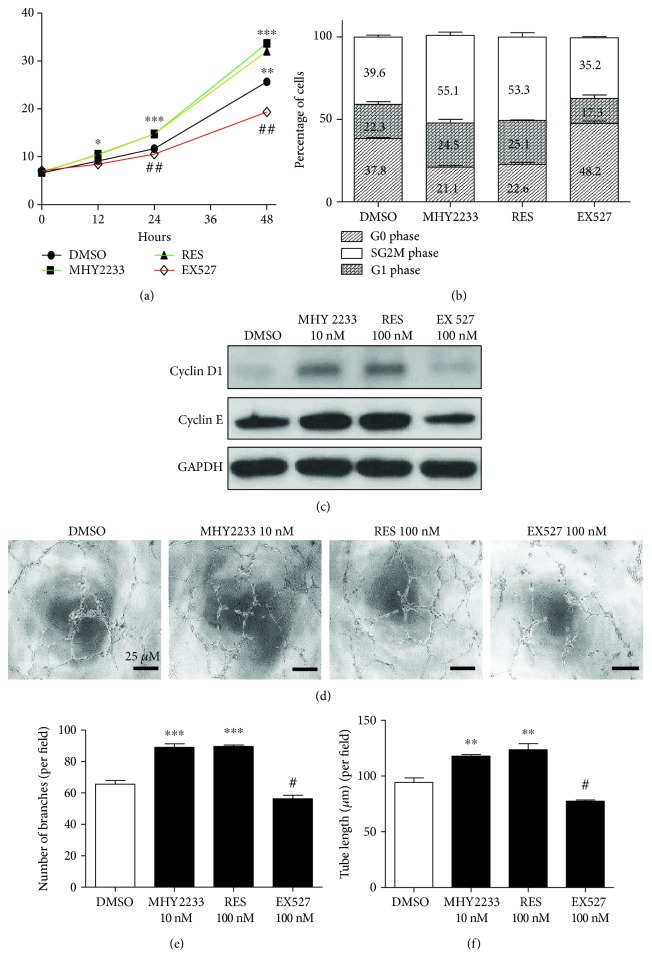
Effect of MHY2233 on cell proliferation and angiogenesis: (a) cell proliferation capacity of senescent EPCs treated with DMSO, 10 nM MHY2233, 100 nM resveratrol, or 100 nM EX527 for 6, 12, 24, and 48 h. After trypsinization, trypan blue unstained cells were counted and plotted. (b) Cell cycle analysis was performed using Hoechst 33342/Pyronin Y and flow cytometry. The percentage of cells in G0, G1, and S/G2/M phases was quantified in senescent EPCs treated with DMSO, 10 nM MHY2233, 100 nM resveratrol, or 100 nM EX527. (c) Cell lysates were used to determine protein expression of cyclin D1 and cyclin E in the various groups by western blot analysis. (d) Representative images of tube-forming capacity of senescent EPCs treated with DMSO, 10 nM MHY2233, 100 nM resveratrol, or 100 nM EX527 on Matrigel®. Scale bars: 25 *μ*M. (e) Quantification of the number of tube branches per field (*n* = 3 per group). (f) Quantification of tube length of branches per field (*μ*M) (*n* = 3 per group). ^#,^^∗^ *p* < 0.05, ^##,^^∗∗^ *p* < 0.005, and ^∗∗∗^ *p* < 0.0005*vs.* DMSO (control) by one-way ANOVA.

To evaluate the effect of MHY2233 on cell proliferation, a cell counting method was used. The cells were collected after treating with trypsin-EDTA and were stained with trypan blue. The unstained cells after 6, 12, 24, and 48 h of MHY2233 treatment were counted. Cell proliferation was enhanced statistically by MHY2233 treatment. The proliferation rate was similar to that with the activator of SIRT1, resveratrol. However, the inhibitor of SIRT1, EX527, decreased cell proliferation when compared with that of DMSO (Figure 5(a)). To determine the reason for the increased cell proliferation, different phases of the cell cycle in different treatment groups were analyzed. MHY2233 decreased the cells in the G0 phase of the cell cycle and increased those in the G1 phase. Moreover, MHY2233 significantly increased the number of cells in the S/G2/M phase of the cell cycle in a similar manner as that of resveratrol. EX527 increased the cells in the G0 phase and decreased those in the G1 and S/G2/M phases of the cell cycle (Figure 5(b)). This was further supported by western blot data of the cell cycle proteins cyclin D1 and cyclin E. MHY2233 and resveratrol increased the protein expression of both cyclins, whereas EX527 decreased their expression when compared with that of DMSO treatment alone (Figure 5(c)). Thus, these data indicate that MHY2233 increases cell proliferation of senescent EPCs by upregulating cell cycle proteins (cyclin D1 and cyclin E) and increasing cell cycle progression.

### 3.6. Effect of MHY2233 on Angiogenesis Potential

**Figure 6:**
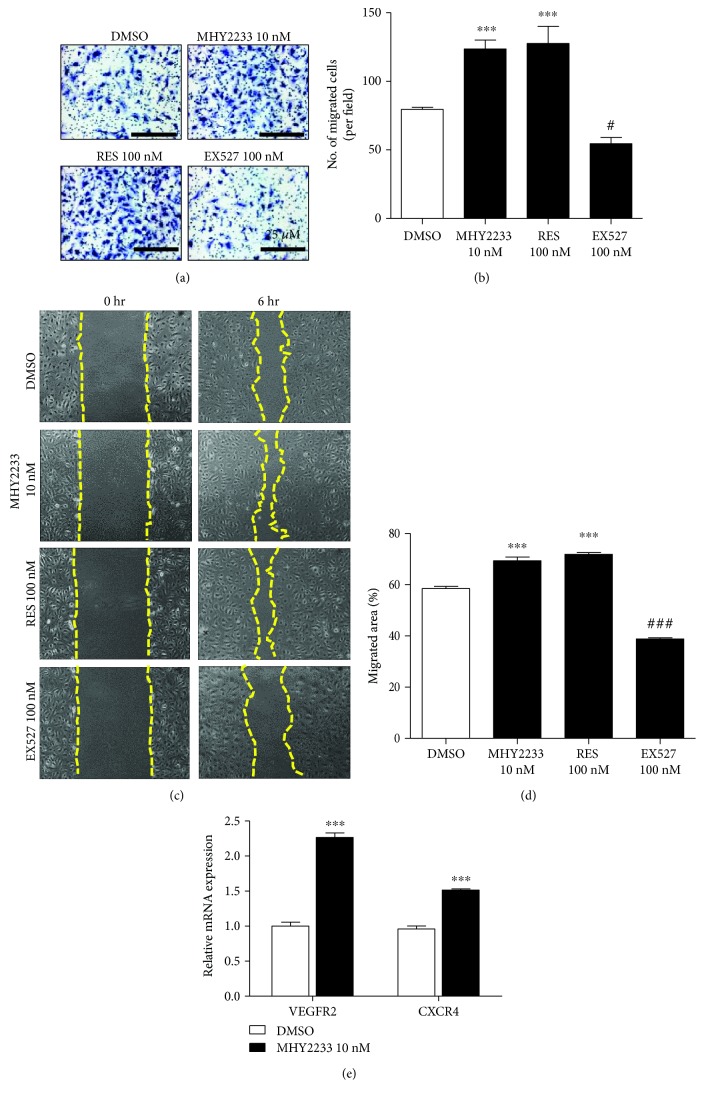
Effect of MHY2233 on cell migration: (a) Transwell® migration assay in senescent EPCs treated with DMSO, 10 nM MHY2233, 100 nM resveratrol, or 100 nM EX527. Cells were stained and examined under a microscope at 100x magnification (*n* = 3 wells per group). Scale bars: 25 *μ*M. (b) Quantification of the number of migrated cells per field in Transwell® using ImageJ software (*n* = 3 per group). (c) Representative images of scratch wound healing migration assay at 0 h and 6 h after scratching. (d) Percentage of migrated area by senescent EPCs in DMSO, 10 nM MHY2233, 100 nM resveratrol, or 100 nM EX527 groups (*n* = 3 per group). (e) Relative mRNA levels of VEGFR2 and CXCR4 in 10 nM MHY2233 group compared with that of DMSO (*n* = 3 per group). ^#^ *p* < 0.05 and ^###,^^∗∗∗^ *p* < 0.0005*vs.* DMSO (control) by one-way ANOVA.

To study the effect of MHY2233 on the functional activities of senescent EPCs, their tube formation capacity on Matrigel®, Transwell® cell migration, and scratch wound healing were assessed. MHY2233 and resveratrol increased the tube-forming capacity, tube length, and number of tube branches formed on Matrigel®. The SIRT1 inhibitor reduced the tube formation, tube length, and number of branches formed (Figures 5(d), 5(e), and 5(f)). Moreover, for Transwell® migration, the number of migrated cells was statistically increased by MHY2233 and resveratrol treatment compared with that of DMSO and was decreased by EX527 treatment (Figures 6(a) and 6(b)). In the scratch wound healing assay, MHY2233 significantly increased the percentage of the area containing migrating cells in a similar manner to that of resveratrol, when compared to that of DMSO. The inhibitor of SIRT1 significantly decreased the migrated area compared to that of control wells (Figures 6(c) and 6(d)). We measured the mRNA level of vascular endothelial growth factor receptor 2 (VEGFR2) and C-X-C chemokine receptor type 4 (CXCR4) and found that MHY2233 increased the mRNA levels of both VEGFR2 and CXCR4 (Figure 6(e)). These increased mRNA levels might be a possible reason for increased migration and tube-forming capacity in senescent EPCs.

**Figure 7:**
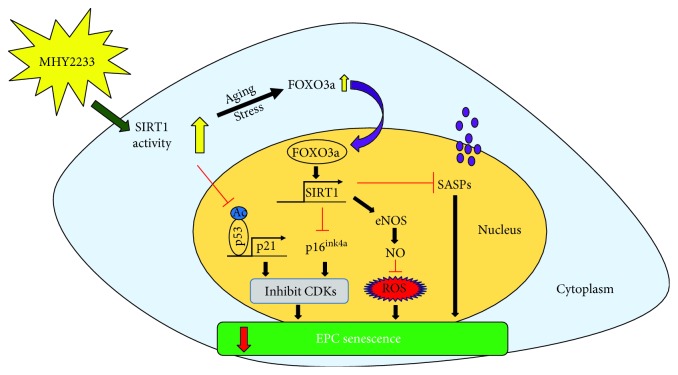
Possible working model of MHY2233 in preventing senescence in EPCs: possible mechanism of MHY2233 to prevent vascular senescence in EPCs. MHY2233 increases SIRT1 activity and, upon aging or stress, increases the activity of FOXO3a, which translocates into the nucleus and upregulates the expression of SIRT1. SIRT1 increases the activity of eNOS and reduces the ROS levels by producing nitric oxide. Similarly, SIRT1 downregulates the SASPs and p16^ink4a^ and reduces EPC senescence. Moreover, SIRT1 activity deacetylates p53 and downregulates the expression of p21, thereby reducing EPC senescence.

In summary, MHY2233 activates SIRT1 activity and upregulates SIRT1 expression. During aging or stress, SIRT1 deacetylase activity increases the activity of FOXO3a, which translocates into the nucleus to increase SIRT1 gene expression. SIRT1 regulates eNOS activity to produce nitric oxide and reduce ROS levels in the endothelium, thus preventing oxidative stress-induced vascular aging. Moreover, SIRT1 blocks the senescence markers, p16^INK4a^, p53, and p21, as well as other SASPs, and aids in the prevention of vascular aging. A schematic diagram that shows a possible mechanism of MHY2233 activity to prevent senescence in EPCs is presented in Figure 7.

## 4. Discussion

This study for the first time demonstrated a new potent SIRT1 activator, MHY2233, to prevent replicative senescence and oxidative stress-induced senescence in cultured human EPCs. The major finding is that MHY2233 not only increases SIRT1 activity but also increases SIRT1 gene expression in EPCs with chronic treatment. Moreover, it reduces the expression of cell cycle arrest proteins (acetylated p53, p16^INK4a^, and p21) and enhances functional activities of senescent EPCs (migration, proliferation, and tube formation capacity). Likewise, MHY2233 decreases ROS formation in senescent EPCs and decreases the percentage of apoptotic cells. Consistently, it reduces cellular ROS with H_2_O_2_-induced oxidative stress, protects EPCs from oxidative stress, and prevents premature senescence. Furthermore, MHY2233 minimizes SA-*β*-gal-positive cells and downregulates SASPs, such as IL-6, IL-8, IL-1*α*, and IL-1*β*, in senescent EPCs. This reveals the potential effect of MHY2233 in preventing vascular senescence.

Aging is a major risk factor for the development of different types of CVDs [19] including atherosclerosis that leads to stroke and myocardial infraction. Various studies have suggested that EPCs show impaired functions and diminished numbers with age [20–22]. Damaged endothelium is an important indicator of aging-related vascular diseases [19, 23]. Among the sirtuin family members, SIRT1 plays an important role especially in endothelial cells for angiogenesis and vasculogenesis [23]. SIRT1 is associated with lifespan extension and other antiaging effects. The SIRT1 activator, SRT1720, increases arterial SIRT1 activity and expression [24]. Donato et al. reported that SIRT1 expression and activity decrease with age in the vasculature of both humans and mice [25]. Likewise, Wang et al. reported that endothelial SIRT1 expression is decreased in senescent EPCs [26, 27]. Our investigation showed that SIRT1 mRNA and protein expression levels are decreased in senescent EPCs.

Previous studies have shown that p53, p16^INK4a^, and p21 are key components of cellular senescence [28]. p21 is a known target of p53, and therefore, p21 expression induced by p53 will inhibit CDKs and mediate cell cycle arrest during senescence [29]. The expression of another CDK inhibitor, p16^INK4a^, also contributes to age-dependent EPC senescence [30]. p53 acetylation promotes the transactivation of many genes controlling cell cycle arrest and apoptosis. SIRT1 inhibits p53 transcription of its downstream effector p21 by deacetylation of p53 at the C-terminal lysine K382 [31, 32]. Moreover, SIRT1 significantly reduces the levels of the CDK inhibitor, p16^INK4a^ [30, 33]. Consistently, this study showed that MHY2233 improved SIRT1 deacetylase activity. SIRT1 activity enhanced the deacetylation of p53 at K382, resulting in low levels of acetylated p53. Moreover, upon chronic treatment with MHY2233, upregulated SIRT1 expression suppressed the p21 expression induced by p53. Likewise, MHY2233 blocked the CDK inhibitor, p16^INK4a^, and prevented cellular senescence in EPCs by activating SIRT1. Stimulation of EPCs with the SIRT1 activator, resveratrol, upregulated SIRT1 expression and downregulated p53 acetylation, thereby blocking the CDK inhibitors, p21 and p16^INK4a^. Moreover, the SIRT1 antagonist, EX527, blocked SIRT1 expression and thus upregulated senescence markers, such as acetylated p53, p21, and p16^INK4a^. Therefore, MHY2233 stimulated SIRT1 expression and prevented EPC replicative senescence.

The mechanism of SIRT1-activating compounds (STACs), such as resveratrol and SRT1720, to increase SIRT1 expression is unclear. There is still controversy whether STACs have a direct or indirect effect on SIRT1 expression [34–36]. SRT1720 [24] and resveratrol were shown to enhance SIRT1 expression and SIRT1 activity [37]. However, other studies suggested that SIRT1 activating compounds (e.g., SRT1720 and resveratrol) are not direct SIRT1 activators [38]. Subsequently, it was confirmed that resveratrol and other STACs activate SIRT1 activity through allosteric enzymatic regulation [34, 39–41]. Among the FOXO subclass isoforms, FOXO1 and FOXO3a are predominant in human endothelial cells [42]. Under stress, FOXO3a translocates into the nucleus [43]. FOXO3a is the target of SIRT1 deacetylase activity [35]. In human cells, FOXO3a interacts with p53 response elements in the promoter region of SIRT1 and increases SIRT1 gene expression upon stress or DNA damage [43–45]. Current results suggest that MHY2233 and resveratrol increase SIRT1 deacetylase activity and, during stress or aging, SIRT1 activity upregulates the expression of FOXO3a. Then, FOXO3a is translocated into the nucleus where it interacts with the proximal promoter of SIRT1 and increases SIRT1 gene transcription. In addition, the inhibitor of SIRT1, EX527, decreases FOXO3a expression and thereby decreases the expression of SIRT1.

Several studies have suggested the role of oxidative stress and ROS in cellular senescence and endothelial dysfunction [8, 46, 47]. Aging of endothelial cells leads to increased ROS formation [48]. Fu et al. reported that stress-induced premature senescence of EPCs caused by oxidative stress was characterized by DNA damage. EPC senescence through the activation of ROS signaling leads to cell cycle arrest. Cyclin D1 is a downstream protein of oxidative stress-induced EPC senescence [49]. ROS, such as superoxide radical, hydroxyl radical, and singlet oxygen, are generally viewed as regulators of aging processes [48]. SIRT1 plays a significant role in oxidative stress resistance *via* SIRT1/eNOS pathways [50]. Resveratrol enhances eNOS gene expression by stimulating endothelial nitric oxide generation [51]. Resveratrol induces eNOS enzymatic activity by enhancing eNOS phosphorylation [42, 52]. The present study showed that MHY2233 protects EPCs from apoptosis caused by ROS. MHY2233 increases eNOS activity through phosphorylation, by activating SIRT1. Furthermore, the SIRT1 antagonist, EX527, decreases the phosphorylation of eNOS. Thus, this study confirmed that MHY2233 can prevent EPC aging caused by oxidative stress and ROS generation by activating the SIRT1/eNOS pathway.

Cellular senescence is the stage where cells can no longer replicate. It is characterized by irreversible cell cycle arrest in the G0/G1 phase. Senescent cells show different morphological characteristics, such as an increase in size, a flattened appearance, and the presence of large vacuoles. Another important characteristic of senescent cells is the expression of SA-*β*-gal [53]. Senescent cells secrete many soluble factors that are called SASPs. IL-6, IL-8, IL-1*α*, and IL-1*β* are key SASPs [53, 54]. The mRNA levels of IL-6 and IL-8 are increased after the induction of senescence. Hayakawa et al. reported that SIRT1 binds to the promoter region of SASP components and protects against aging by suppressing SASP expression through epigenetic gene regulation [54]. Likewise, our study indicates that the SA-*β*-gal enzyme and SASPs, such as IL-6, IL-8, IL-1*α*, and IL-1*β*, were upregulated in senescent EPCs. Upon treatment with MHY2233, there were significant reductions in the expression of SASPs and the number of SA-*β*-gal-positive cells. This suggests that MHY2233 prevents senescence in EPCs by stimulating SIRT1.

Several previous studies mentioned that the functional activities of EPCs (e.g., cell proliferation, cell migration, and tube formation) were reduced in replicative aging or cellular senescence [26, 55]. Our results also demonstrated that senescent EPCs had impaired functions, namely, proliferation, migration, and angiogenesis. Li et al. reported that SIRT1 promoted EPC migration and proliferation by activation of eNOS expression [56]. SIRT1 promotes angiogenesis by increasing vascular endothelial growth factor (VEGF) expression [26]. In the present study, MHY2233 and resveratrol upregulated eNOS activity in senescent EPCs and therefore increased angiogenesis and cell migration. Moreover, MHY2233 upregulated the expression of the cell cycle proteins, cyclin D1 and cyclin E, stimulated progression into the S/G2/M phase of the cell cycle, and promoted cell proliferation in senescent EPCs. In addition, MHY2233 increased the mRNA levels of VEGFR2 and CXCR4, thus enhancing the angiogenic capacity of senescent EPCs. Treatment with the SIRT1 antagonist, EX527, decreased the cell proliferation, migration, and tube-forming capacity of EPCs. Thus, collectively, it can be concluded that MHY2233 increases the functional activities of EPCs by activating the SIRT1/eNOS pathway and upregulating cyclin D1 and cyclin E protein levels.

## 5. Conclusions

In conclusion, to the best of our knowledge, this is the first study to demonstrate the role of MHY2233 in preventing senescence in human endothelial progenitor cells. The antiaging effect of MHY2233 is mainly due to SIRT1 deacetylase activity and upregulation of SIRT1 expression. MHY2233 was found to be highly effective in reducing replicative senescence and oxidative stress-induced senescence in EPCs. Moreover, MHY2233 increases the functional activities of senescent EPCs, such as cell proliferation, migration, and angiogenesis. This study reveals that MHY2233 is more potent than the commercially available SIRT1 activator, resveratrol. Therefore, the novel compound MHY2233 can be used as a potent therapeutic target for the treatment of aging- and age-associated cardiovascular diseases.

## Figures and Tables

**Table 1:** List of primer sequences used for qRT-PCR.

Name of gene (human)	Sequences of primers used
GAPDH	Forward: ACCACAGTCCATGCCATCAC
	Reverse: TCCACCACCCTGTTGCTGT
SIRT1	Forward: GCAGATTAGTAGGCGGCTTG
	Reverse: TCTGGCATGTCCCACTATCA
SIRT2	Forward: CCGGCCTCTATGACAACCTA
	Reverse: GGAGTAGCCCCTTGTCCTTC
SIRT3	Forward: CAGTCTGCCAAAGACCCTTC
	Reverse: AAATCAACCACATGCAGCAA
SIRT4	Forward: CAGCAAGTCCTCCTCTGGAC
	Reverse: CCAGCCTACGAAGTTTCTCG
SIRT5	Forward: GCTCGCCCACTGTGATTTAT
	Reverse: CCCTGGAAATGAAACCTGAA
SIRT6	Forward: CCAAGTTCGACACCACCTTT
	Reverse: CGGACGTACTGCGTCTTACA
SIRT7	Forward: CGCCAAATACTTGGTCGTCT
	Reverse: GTGATGCTCATGTGGGTGAG
p16	Forward: CCCCGATTGAAAGAACCAGAGA
	Reverse: ACGGTAGTGGGGGAAGGCATAT
p21	Forward: CCGCCCCCTCCTCTAGCTGT
	Reverse: CCCCCATCATACCCCTAACACA
p53	Forward: CCGGCGCACAGAGGAAGAGA
	Reverse: TGGGGAGAGGACTGGTGTTGT
IL-6	Forward: TACCCCCAGGAGAAGATTCC
	Reverse: TTTTCTGCCAGTGCCTCTTT
IL-8	Forward: GTGCAGTTTTGCCAAGGAGT
	Reverse: CTCTGCACCCAGTTTTCCTT
IL-1*α*	Forward: AATGACGCCCTCAATCAAAG
	Reverse: TGGGTATCTCAGGCATCTCC
IL-1*β*	Forward: GGGCCTCAAGGAAAAGAATC
	Reverse: TTCTGCTTGAGAGGTGCTGA
VEGFR2	Forward: GTGACCAACATGGAGTCGTG
	Reverse: TGCTTCACAGAAGACCATGC
CXCR4	Forward: ATGCAAGGCAGTCCATGTCA
	Reverse: ATGAATGTCCACCTCGCTTT

## Data Availability

The data used to support the findings of this study are included in the article and within the supplementary file.

## Supplementary Materials

Supplementary Materials Supplementary Figure S1: mRNA levels of SIRT1, p53, p21, and p16 in young and senescent EPCs were determined using qRT-PCR (*n* = 3 per group). ^∗^ *p* < 0.05 and ^∗∗^ *p* < 0.005 vs. young EPCs by unpaired Student's *t*-test. Supplementary Figure S2: EPCs were seeded in 96-well plates and treated with different concentrations of resveratrol and EX527 for 24 h (*n* = 6 per group). (A) Cytotoxicity assay of resveratrol and (B) cytotoxicity assay of EX527 determined using WST-8 assay (*n* = 6 per group). ^∗^ *p* < 0.05, ^∗∗^ *p* < 0.005, and ^∗∗∗^ *p* < 0.0005 vs. DMSO (control) by a one-way ANOVA test. Supplementary Figure S3: relative mRNA levels of (A) SIRT1, (B) p16, (C) p53, and (D) p21 in senescent EPCs treated with MHY2233 in a concentration-dependent manner (10 nM, 100 nM, and 1 *μ*M) using qRT-PCR (*n* = 3 per group). ^∗^ *p* < 0.05, ^∗∗^ *p* < 0.005, and ^∗∗∗^ *p* < 0.0005 vs. DMSO (control) by a one-way ANOVA test. Supplementary Figure S4: representative confocal images of immunofluorescence staining for SIRT1, p16, ac-p53, and p21 (red) in senescent EPCs treated with DMSO or 10 nM MHY2233 (*n* = 3). The nuclei were stained with DAPI (blue). Scale bars 20 *μ*M.
