# Supplementary material for: MHY2233 Attenuates Replicative Cellular Senescence in Human Endothelial Progenitor Cells via SIRT1 Signaling
Source: Oxid Med Cell Longev. 2019 May 22;2019:6492029. doi: 10.1155/2019/6492029 (PMC6556284; doi:10.1155/2019/6492029)
Supplement: Supplementary Materials — Supplementary Figure S1: mRNA levels of SIRT1, p53, p21, and p16 in young and senescent EPCs were determined using qRT-PCR (n = 3 per group). ∗ p < 0.05 and ∗∗ p < 0.005 vs. young EPCs by unpaired Student's t-test. Supplementary Figure S2: EPCs were seeded in 96-well plates and treated with different concentrations of resveratrol and EX527 for 24 h (n = 6 per group). (A) Cytotoxicity assay of resveratrol and (B) cytotoxicity assay of EX527 determined using WST-8 assay (n = 6 per group). ∗ p < 0.05, ∗∗ p < 0.005, and ∗∗∗ p < 0.0005 vs. DMSO (control) by a one-way ANOVA test. Supplementary Figure S3: relative mRNA levels of (A) SIRT1, (B) p16, (C) p53, and (D) p21 in senescent EPCs treated with MHY2233 in a concentration-dependent manner (10 nM, 100 nM, and 1 μM) using qRT-PCR (n = 3 per group). ∗ p < 0.05, ∗∗ p < 0.005, and ∗∗∗ p < 0.0005 vs. DMSO (control) by a one-way ANOVA test. Supplementary Figure S4: representative confocal images of immunofluorescence staining for SIRT1, p16, ac-p53, and p21 (red) in senescent EPCs treated with DMSO or 10 nM MHY2233 (n = 3). The nuclei were stained with DAPI (blue). Scale bars 20 μM. [file 6492029.f1.docx]

**
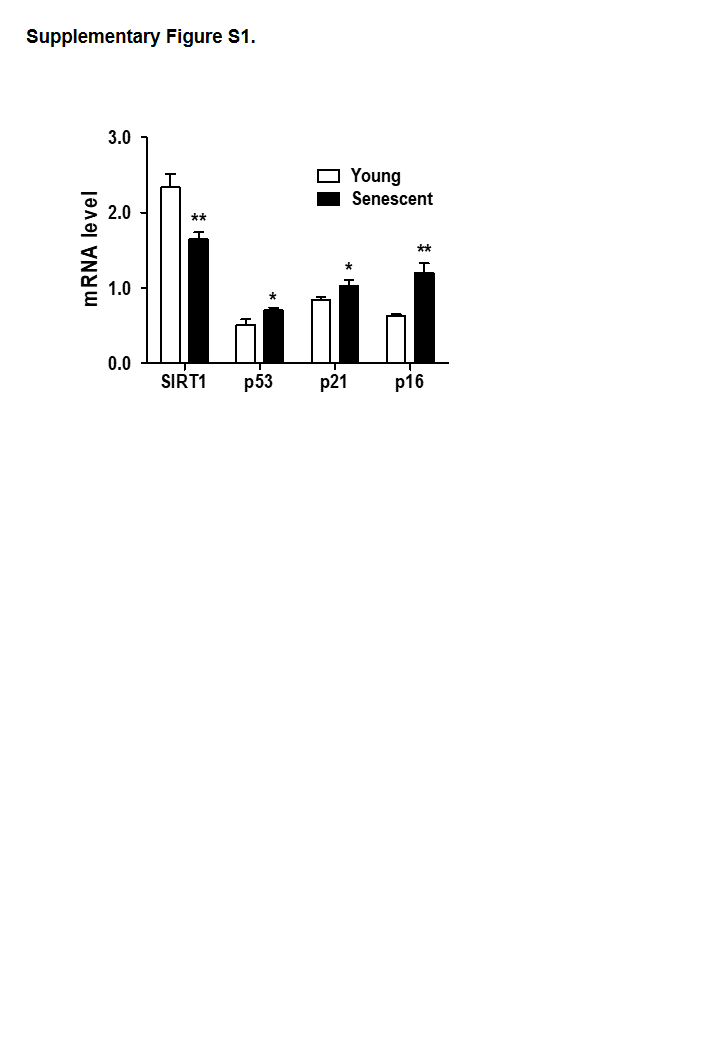
**

**Supplementary Figure S1**

mRNA levels of SIRT1, p53, p21, and p16 in young and senescent EPCs were determined using qRT-PCR, *n*=3 per group. **p*<0.05 and ***p*<0.005 *vs.* young EPCs by unpaired Student’s *t* test.

**
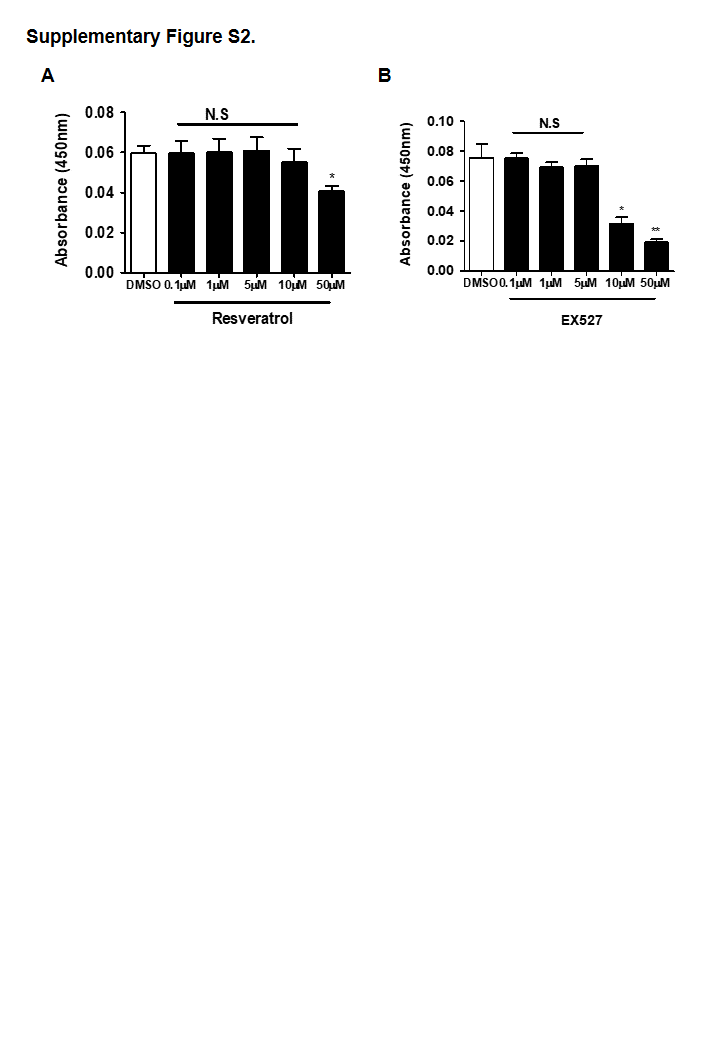
**

**Supplementary Figure S2**

EPCs were seeded in 96-well plates and treated with different concentrations of resveratrol and EX527 for 24 h, *n*=6 per group. (A) Cytotoxicity assay of resveratrol and (B) Cytotoxicity assay of EX527 determined using WST-8 assay, *n*=6 per group. **p*<0.05, ***p*<0.005, and ****p*<0.0005 *vs.* DMSO (Control) by one-way ANOVA test.

**
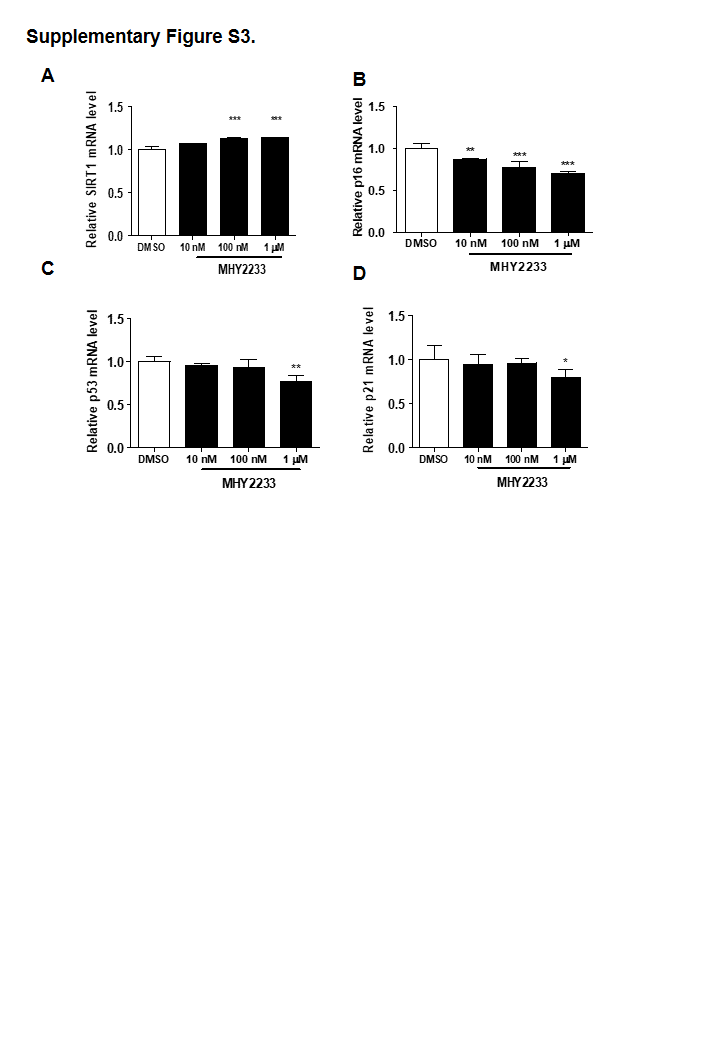
**

**Supplementary Figure S3**

Relative mRNA levels of (A) SIRT1, (B) p16, (C) p53, and (D) p21 in senescent EPCs treated with MHY2233 in a concentration-dependent manner (10 nM, 100 nM, and 1 µM) using qRT-PCR, *n*=3 per group. **p*<0.05, ***p*<0.005, and ****p*<0.0005 *vs.* DMSO (Control) by one-way ANOVA test.


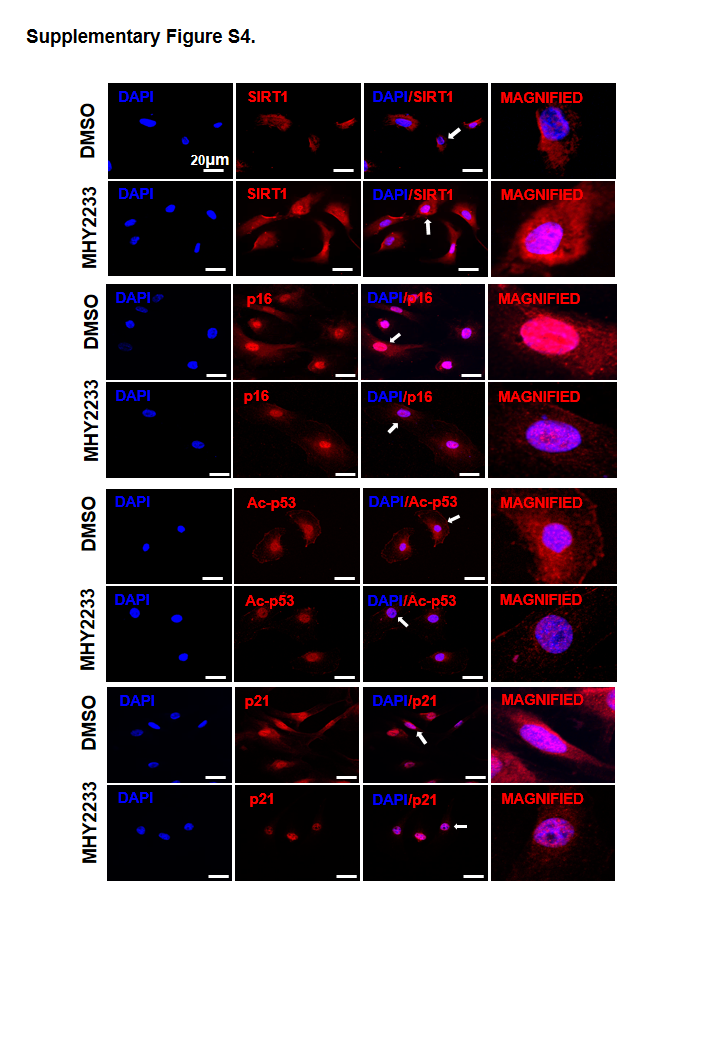
**Supplementary Figure S4**

Representative confocal images of immunofluorescence staining for SIRT1, p16, ac-p53, and p21 (red) in senescent EPCs treated with DMSO or 10 nM MHY2233; *n*=3. The nuclei were stained with DAPI (blue). Scale bars 20 µm.
